# A unified framework for the organization of the primate auditory cortex

**DOI:** 10.3389/fnsys.2013.00011

**Published:** 2013-04-30

**Authors:** Simon Baumann, Christopher I. Petkov, Timothy D. Griffiths

**Affiliations:** Medical Faculty, Institute of Neuroscience, Newcastle UniversityNewcastle upon Tyne, UK

**Keywords:** auditory cortex, primate, humans, tonotopy, anatomy, comparative

## Abstract

In non-human primates a scheme for the organization of the auditory cortex is frequently used to localize auditory processes. The scheme allows a common basis for comparison of functional organization across non-human primate species. However, although a body of functional and structural data in non-human primates supports an accepted scheme of nearly a dozen neighboring functional areas, can this scheme be directly applied to humans? Attempts to expand the scheme of auditory cortical fields in humans have been severely hampered by a recent controversy about the organization of tonotopic maps in humans, centered on two different models with radically different organization. We point out observations that reconcile the previous models and suggest a distinct model in which the human cortical organization is much more like that of other primates. This unified framework allows a more robust and detailed comparison of auditory cortex organization across primate species including humans.

## Introduction

One of the oldest and best characterized organizational features in the auditory system is its cochleotopic or tonotopic organization. Tonotopy is the ordered representation of sound frequency in auditory areas. It has been shown at all levels of the auditory pathway including the cochlea, the auditory brainstem nuclei, and the auditory cortex in at least mammals and birds. In the cortex of non-human primates, multiple areas can be defined neurophysiologically by gradients of neuronal sound frequency preference and by reversals of the frequency gradient between neighboring auditory cortical areas (Hackett et al., 1998; Kaas and Hackett, 2000; Hackett, 2011). More recently, non-invasive imaging using functional MRI (fMRI) has defined mirror-symmetric tonotopic gradients that allow division of the auditory cortex of non-human primates (Petkov et al., 2006; Baumann et al., 2010; Tanji et al., 2010) and humans (for a recent review see: Woods and Alain, 2009) into distinct areas.

Given the broadly conserved role of tonotopy in the auditory system of mammals, it is surprising that in all of the investigated species the organization of tonotopy in the human auditory cortex is one of the least understood. This is in spite of several decades of neuroimaging studies and efforts to understand the human auditory cortical organization. Moreover, even after a recent resurgence in human tonotopic studies we seem to be getting further from an agreed model of human auditory cortical organization. Although a considerable body of anatomical data appear to support an organization of the primary auditory (core) areas along the length of Heschl's gyrus (HG; Brodmann, 1909; von Economo and Koskinas, 1925; von Economo and Horn, 1930; Galaburda and Sanides, 1980; Rivier and Clarke, 1997; Morosan et al., 2001, 2005; Wallace et al., 2002), for which early studies of tonotopy appeared to provide support (Ojemann, 1983; Lauter et al., 1985; Howard et al., 1996; Wessinger et al., 1997; Bilecen et al., 1998; Lockwood et al., 1999; Talavage et al., 2000; Schonwiesner et al., 2002; Formisano et al., 2003), recent studies of tonotopy can be interpreted in terms of a fundamentally different organization of these core areas (Humphries et al., 2010; Woods et al., 2010; Da Costa et al., 2011; Striem-Amit et al., 2011; Langers and van Dijk, 2012). In this, the mirror-symmetric gradients in the auditory core is effectively perpendicular to the one suggested by anatomical and early functional studies. This has led to a fundamental reappraisal of the homology between non-human and human auditory areas.

Here, we critically examine studies of non-human and human primates that led to the traditional view of tonotopic field organization in the human auditory cortex. This view is based on a tonotopic axis that runs along the HG, the most characteristic anatomical feature in the human auditory cortex. We contrast this view with more recent interpretations based on a tonotopic axis that runs perpendicular to the HG. Taking all the current evidence from human but also non-human primates in account, we propose a unifying interpretation. This reconciles the apparently conflicting evidence supporting both previous views, and emphasizes the striking similarity of the human tonotopic maps to those in non-human primates.

## Tonotopic organization of cortical fields in non-human primates

**Figure 1:**
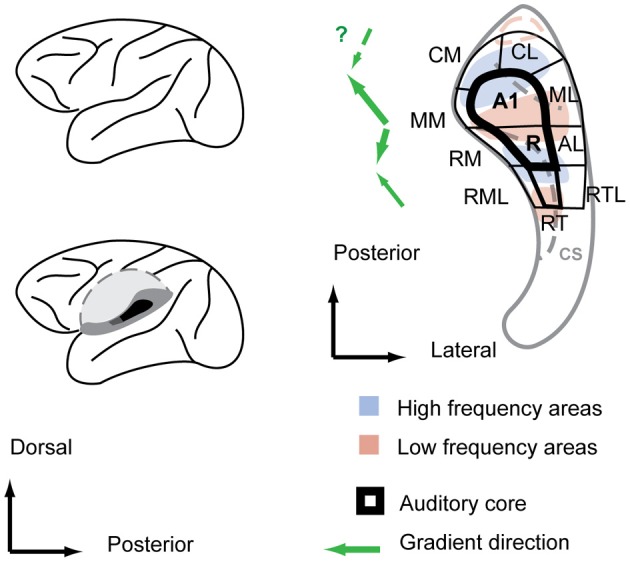
**Functional field organization of the monkey auditory cortex superimposed over the frequency response areas and anatomical features of the macaque superior temporal plane. Left:** a sagittal view of the macaque brain (top) with the superior temporal plane (dark gray shadings) exposed by cutting out the operculum (bottom). **Right:** within the superior temporal plane, the core area, highlighted with bold stroke, is surrounded by belt fields. Anatomical features are designated by light gray color shade. Main gradient directions from low to high of the frequency response areas are indicated with green arrows left of the scheme. CS, circular sulcus. Auditory field labeling according to Hackett et al. (Hackett et al., 1998; Hackett, 2011).

As in other mammals, electrophysiological studies in non-human primates have established tonotopically organized areas at multiple levels of the auditory pathway including the inferior colliculus (IC) (Ryan and Miller, 1978; Zwiers et al., 2004), the medial geniculate body (MGB) (Gross et al., 1974), and the auditory cortex (Merzenich and Brugge, 1973; Morel et al., 1993; Kosaki et al., 1997; Rauschecker et al., 1997; Bendor and Wang, 2008). More recently, the existence and detailed organization of tonotopic fields has been confirmed by fMRI in the monkey IC (Baumann et al., 2011) and auditory cortex (Petkov et al., 2006; Baumann et al., 2010; Tanji et al., 2010). Cytoarchitectonic mapping, histochemical- and anterograde staining studies in the primate auditory cortex have established a concentric organization of auditory core areas that receive input mainly from an auditory pathway via the central nucleus of the IC (ICc) and the ventral portion of the MGB (MGv) and surrounding belt areas that receive input mainly from a distinct auditory pathway via the dorsal and lateral cortices of the IC and the dorsal MGB (MGd) [reviewed in Hackett (2007b)]. An influential organizational scheme (Hackett et al., 1998; Kaas and Hackett, 2000; Hackett, 2011) combined anatomical and functional data and subdivided core and belt areas into 2–3 core fields (primary-like fields) and 7–8 belt fields based on reversals of the tonotopic gradients running along a largely anterior-posterior axis (Figure 1). The scheme suggests that gradient reversals to mark functional area borders are a fundamental feature of cortical sensory organization analog to the visual cortex system where retinotopic gradient reversals mark functional area borders (Gattass et al., 1988; Sereno et al., 1995).

The tonotopic gradients are most obvious in the core fields A1 and R. In the macaque, the high to low frequency gradient of A1 starts typically in the midline of the posterior superior temporal plane and runs antero-laterally to the cusp of the circular sulcus (Merzenich and Brugge, 1973). A slight protuberance in the superior temporal plane at the cusp of the circular sulcus is a consistent anatomical predictor of the location of the low frequency reversal (Baumann et al., 2010). From there, the reversed low-to-high tonotopic gradient of R typically turns slightly inward and covers the slope into the circular sulcus and part of its depth. Consequently, the tonotopic axis of the two main core fields A1 and R is not straight but features a considerable kink (Morel et al., 1993; Kosaki et al., 1997; Kaas and Hackett, 2000; Baumann et al., 2010), which has also been seen in marmosets (Bendor and Wang, 2008). A number of monkey studies (Morel and Kaas, 1992; Petkov et al., 2006; Bendor and Wang, 2008; Baumann et al., 2010; Tanji et al., 2010) report a further gradient reversal in the depth of the circular sulcus, an area that is designated RT in the Hacket and Kaas scheme (Hackett et al., 1998). However, there has been inconsistency in the earlier data on which way the tonotopic gradient would run in RT (Morel and Kaas, 1992). More recent monkey fMRI studies, however, consistently identify RT with a mirror reversed gradient in relation to the more posterior field R (Petkov et al., 2006; Baumann et al., 2010; Tanji et al., 2010). A microelectrocorticographic study in macaques showed that mirror-symmetric tonotopic gradients in the fields A1, R, and RT are even detectable in the spatial covariation of spontaneous activity in the absence of stimuli (Fukushima et al., 2012). Histochemical markers for core areas such as parvalbumin, myelin, and acetylcholinesterase density show values for RT that are somewhat between the typical values for auditory core and auditory belt areas (Hackett et al., 1998), and it features a cell packing density closer to belt areas (Hackett et al., 2001), thus it is also referred to as “core-like” area rather than core proper (Hackett et al., 2001).

The tonotopic gradients of the core fields extend into the adjacent belt fields (Rauschecker et al., 1995; Kosaki et al., 1997). Thus, the reversals of the tonotopic gradients can provide the anterior/posterior borders between the belt fields similar to the core (Hackett et al., 1998) (Figure 1). For instance, monkey fMRI studies (Petkov et al., 2006; Baumann et al., 2010) have delineated most of the belt fields including some for which there was only structural evidence. Nevertheless, the tonotopic gradients in the belt areas tend to be less robust than the core areas since the belt fields respond weakly to tones and responses to band-passed noise can elicit responses in a larger area making the determination of borders more difficult. While tonotopic arrangements beyond the belt fields (e.g., in the adjacent parabelt) requires further investigation, the tonotopic organization of the auditory core and belt fields in non-human primates is generally uncontroversial to date.

## Organization of the human auditory cortex—the classical configuration

Before the advent of non-invasive imaging methods, functional studies from the human auditory cortex were limited [see Ojemann (1983) and Howard et al. (1996)]. For most of the 20th century post-mortem anatomical studies provided the only clues on the organization of the human auditory cortex. Beginning with Brodmann (1909), a number of studies mapped cytoarchitectonically distinctive fields in the human auditory cortex (von Economo and Koskinas, 1925; Beck, 1928; von Economo and Horn, 1930; Galaburda and Sanides, 1980; Rivier and Clarke, 1997; Morosan et al., 2001, 2005; Wallace et al., 2002) [reviewed in Hackett (2007a), Figure 2]. The different studies vary in the nomenclature and the number of identified auditory fields. However, the different studies share several findings. All show one (or several adjacent) fields in the central part of HG, which are called koniocortex because of a well-developed layer 4 characteristic of primary sensory cortex. The koniocortex is similar in structure to the core areas of the non-human primate in the scheme above (Hackett et al., 1998) (Figure 1). This core region is surrounded by areas that are closer in terms of cytoarchitechtonics to the belt areas of other mammals. Hence, cytoarchitecture suggests an auditory cortex organization in humans that is similar to the non-human primate core and belt model. In this overall organization the studies mainly differ in the extent of HG that the core is covering. In the majority of studies the core is restricted to the central portion of the HG and the most medial and the most lateral portion is attributed to the belt (von Economo and Koskinas, 1925; von Economo and Horn, 1930; Galaburda and Sanides, 1980; Rivier and Clarke, 1997; Morosan et al., 2001, 2005). However, in some of the studies (Brodmann, 1909; Beck, 1928) the core also extends to the most medial part of the HG or even encompasses the entire HG (Brodmann, 1909). Of particular relevance to the recent controversy about the location and direction of the main axis of the tonotopic areas are the anterior and posterior borders of the auditory core areas. While most newer studies based on cytoarchitecture (Beck, 1928; Galaburda and Sanides, 1980; Rivier and Clarke, 1997; Morosan et al., 2001) suggest core areas that are within the anterior and posterior limits of HG, the core areas in Brodmann (1909), von Economo and Koskinas (1925), and von Economo and Horn (1930) are more extensive and stretch clearly beyond the HG to part of the planum temporale (PT) situated immediately behind.

**Figure 2:**
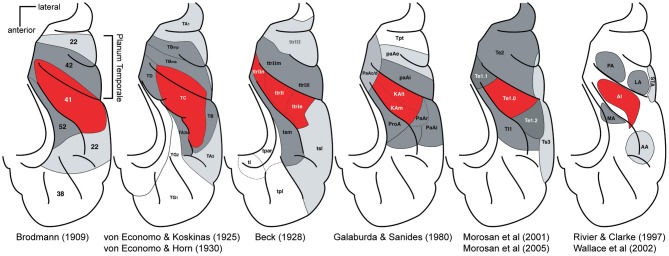
**Parcelations of the human superior temporal cortex by different investigators.** For each panel, the locations of major auditory cortical regions are drawn on a standardized schematic of the superior temporal plane. The STG is not visible. Red, core region; dark gray, belt region, light gray parabelt, and possibly other regions. Posterior is up, lateral is right. Adapted by Troy Hackett from Hackett (2007a,b).

Although these cytoarchitectonic studies showed differences in the precise borders of the primary or core area with respect to HG [this is emphasized in a further multi-subject study (Rademacher et al., 2001)], HG became established early on as an easy-to-identify marker for the location of the core areas in humans. Thus, when non-invasive neuroimaging methods allowed for the first time detailed functional investigations in the human auditory cortex, the direction of the tonotopic gradient along the HG seemed to be the main question that needed to be resolved. There was little expectation that the main tonotopic axis would not be in HG. The similarity of the elongated shape of HG with the elongated (but differently orientated) non-human primate core area likely provided another strong bias.

Early Positron Emission Tomography (PET) and fMRI did not provide the resolution and power for detailed tonotopic maps in the auditory cortex. However, a number of PET and fMRI studies consistently demonstrated significantly activated clusters or voxels responding to high frequency tones in the vicinity of the medial HG and to low frequency tones in the lateral HG (Lauter et al., 1985; Wessinger et al., 1997; Bilecen et al., 1998; Lockwood et al., 1999; Talavage et al., 2000). Given the previous anatomical evidence for a primary auditory area in the HG and the fact that homologous areas in other mammals are tonotopically organized, the common interpretation of these early imaging studies was that the HG contained a single tonotopic gradient progressing in antero-lateral direction from high frequencies in the medial HG along the gyrus to low frequencies in the lateral portion. This tonotopy configuration in humans was, with minor modifications, widely accepted. We will call this model here the “classical configuration” of human auditory cortex organization.

**Figure 3:**
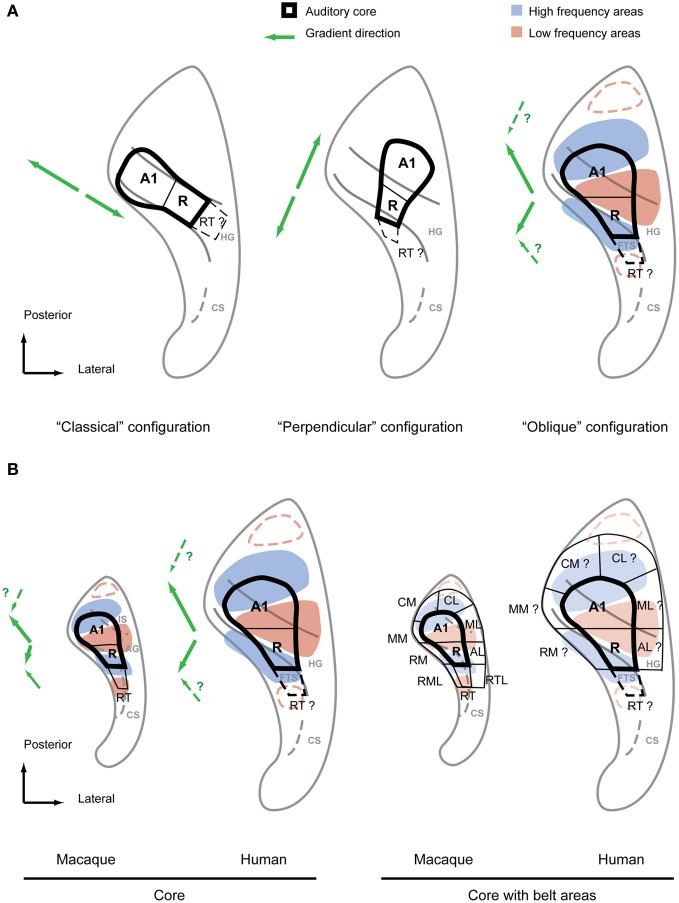
**Debated configurations of auditory cortical organization in humans and non-human primate configuration for comparison. (A)** The two main configurations of auditory core fields under debate (left, middle) in comparison with the “oblique” configuration proposed by the authors (right). The main frequency response areas based on the summary of recent evidence (Formisano et al., 2003; Humphries et al., 2010; Woods et al., 2010; Da Costa et al., 2011; Striem-Amit et al., 2011; Langers and van Dijk, 2012) are superimposed over this configuration. The suggested directions of the main gradient axes are indicated with green arrows next to each configuration. Additional anterior and posterior low frequency preference areas suggested by some studies are marked by red dashed lines. **(B)** Core fields and frequency preference areas in the superior temporal plane of macaque and human according to oblique configuration (left). Location of auditory belt fields in macaques and presumed location of belt fields in humans (right). Main gradient directions from low to high of the frequency response areas are indicated with green arrows left of each scheme. IS, intercalated sulcus; AG, annectant gyrus; CS, circular sulcus; FTS, first transversal sulcus.

In 2002 some fMRI workers (Schonwiesner et al., 2002) already questioned whether human tonotopic organization might be more complex than the classical configuration suggested, despite finding very similar activation patterns to the previous imaging studies. In contrast to previous studies a range of tones at frequencies from 0.25 to 8 kHz were presented in this work. The derived response pattern did not form a simple and continuous gradient along the HG that could be easily compared to the primate model, nor could the response pattern be reconciled with the multiple mirror-symmetric tonotopy gradients from the data in non-human primates. A year later a study performed at high magnetic field with high resolution (seven Tesla MRI field-strength) (Formisano et al., 2003) seemed to answer all these questions. This high resolution dataset was derived from six subjects presented with six tones of different frequency, which was analyzed using flat-mapping techniques and a gradient analysis similar to those used in visual system (Van Essen and Zeki, 1978; Sereno et al., 1995). This study also represented the three dimensional curvature of the auditory cortex in a plane from rendered brains. The results were detailed, planar maps covering the central part of the auditory cortex with several high-frequency clusters that progressed in a continuous gradient into low-frequency areas. In one subject (Figure 3 in the report) the direct line between two high frequency maxima was approximately located in the medial two thirds of the HG and, by crossing a low-frequency maxima, marked two mirror-symmetric gradients (high-low-high) roughly in the direction of the long axis of the HG. Further subjects (Figure 5 in report) showed a similar although slightly more complex pattern without such precise alignment of the tonotopic axis along the length of HG. These results were not only compelling because they strikingly differed in their detail and completeness from previous studies but they also fulfilled the expectation of a monkey-like, mirror-symmetric tonotopic gradient reversal along HG representing hA1 and hR, potential human homologs of the monkey core areas A1 and R. Furthermore, this interpretation allowed room for a third gradient reversal to be detected representing the area RT in the lateral HG. Hence this study provided an updated “classical configuration” that still featured a tonotopic axis along the HG but accommodated all the core fields known from non-human primates with at least one gradient reversal instead of single tontopic gradient (see Figure 3A, left). Although a further detailed study in (Talavage et al., 2004) suggested a more complex arrangement including a number of tonotopic axes that differed in location and direction from those of Formisano et al. (2003), this classical configuration remained widely accepted until recently.

## Reorienting the gradient axis in humans—the “perpendicular configuration”

The classical tonotopy configuration was first fundamentally challenged by Humphries et al. (2010). This study used flat mapping of the fMRI responses to multiple narrowband stimuli, as in Formisano et al. (2003) and Talavage et al. (2004). However, Humphries et al. (2010) proposed two principal mirror-symmetric tonotopic gradients with an axis running in a postero-lateral to antero-medial direction formed by high frequency areas posterior to HG (in the PT) and anterior to HG separated by a low frequency area on the crest of the HG. It was suggested that the posterior gradient represented a homologous field to A1 in non-human primates whilst the anterior gradient represented a homologous field to R. According to this proposal, a human homolog of the monkey core area would cross the HG perpendicularly in antero-medial orientation (Figure 3A, middle). Thus, we call the proposed configuration the “perpendicular configuration.” Compared to the previous classical configuration with a tonotopic axis along HG this new model essentially proposes an orthogonal tonotopic axis direction.

The perpendicular configuration of tonotopy stands not only in fundamental contrast to the classical configuration but it also deviates considerably from anatomical studies suggesting that koniocortex co-localizes with HG (Figure 2). The study of Humphries et al. (2010) has been followed by several tonotopy studies (Woods et al., 2010; Da Costa et al., 2011; Striem-Amit et al., 2011; Langers and van Dijk, 2012) in an effort to settle the question of tonotopic field arrangement. A number of methodological refinements have yielded maps of considerable detail and clear frequency specificity with a continuous progression between high- and low-frequency fields within the superior temporal plane. All of these studies showed at least two mirrorsymmetric gradients in the center of the auditory cortex. They also suggested a tonotopic axis that crossed the HG either perpendicularly (Striem-Amit et al., 2011) or in more oblique, diagonal orientation along a posterior-anterior axis direction (Woods et al., 2010; Langers and van Dijk, 2012) with an anterior gradient that deviates medially (Da Costa et al., 2011; Langers and van Dijk, 2012). Da Costa et al. (2011) even considered the possibility of a posterior gradient in the postero-medial to antero-lateral direction. Thus, although more recent studies support Humphries et al.'s (2010) suggestion of high frequency areas posterior and anterior of the HG, there was also a considerable variation in the details of the proposed gradients. In some instances, e.g., the mentioned posterior gradient of Da Costa et al. (2011), aspects of the recent studies where actually closer to Formisano et al.'s (2003) suggestion of a gradient along HG. Furthermore, since these new studies agreed with Humphries et al. (2010) in the assignment of the posterior gradient to an A1 homolog and the anterior gradient to an R homolog, they are similarly at odds with the previous cytoarchitectonic studies which suggested an auditory core area co-localized with the HG. All the tonotopy studies (Woods et al., 2010; Da Costa et al., 2011; Striem-Amit et al., 2011; Langers and van Dijk, 2012) since Humphries et al. (2010) suggested tonotopic gradients or corresponding core fields that extend significantly posterior to HG into the PT and anterior to HG into the first transversal sulcus (FTS). How can the different interpretations of these functional imaging studies be reconciled with each other, and with the data from anatomical studies?

## A unified model of primate auditory cortex representation: what can we learn from the monkey?

Irrespective of different experimental details and despite the variety of interpretations of gradient directions, the recent studies (Formisano et al., 2003; Humphries et al., 2010; Woods et al., 2010; Da Costa et al., 2011; Striem-Amit et al., 2011; Langers and van Dijk, 2012) show a surprisingly consistent pattern of preferred frequency response areas. If we focus on the large scale pattern of preferred frequency responses, dominant high frequency areas posterior and anterior to HG become obvious with a dominant low frequency area in the HG as originally suggested by Humphries et al. (2010). However, these preferred frequency areas do not simply form parallel stripes of alternating high and low frequency preference zones nor is the HG necessarily aligned with these stripes as Humphries et al. (2010) implied. Rather, we find that the posterior and anterior high frequency areas are in closer proximity to each other on the medial side than on the lateral side, almost joining in some subjects on the medial HG. This “V” or “U” shaped pattern formed by the high frequency areas provides space for a roughly wedge-shaped low frequency area on the lateral HG (Figure 3A, right). Although the macrostructure of the HG does not strictly separate high and low frequency areas, it is nevertheless predictably positioned relative to the low-frequency region. Apart from the low frequency area on the lateral HG [or between HG1 and HG2 if a duplication occurs (Da Costa et al., 2011)], the location and orientation of the anterior high frequency area is reliably predicted by the first transverse sulcus that confines HG on its anterior side. Finally, the posterior high-frequency area consistently extends on the lateral side beyond the posterior bank of HG into the PT (even in the case of a HG duplication) but often overlaps with the HG on the medial side. This common frequency response pattern with a predictable relationship to the macro-anatomy (illustrated in Figure 3) is particularly obvious in the group data of the different studies. It is not surprising that the inter-individual variability of the tonotopic pattern is higher in humans than in non-human primates given the more pronounced and variable gyrification in humans. Adaptation to speech is a further possible driving force to functional reorganization and variability. Nevertheless, the general aspects of this pattern are well discernible in both hemispheres even at the individual subject level.

Retrospectively, the frequency response pattern outlined based on recent studies is also consistent with the results from Formisano et al. (2003) and even the preceding imaging studies. While the V-shape pattern is probably most obvious in Figure 5B of Formisano et al. (2003), Figure 3 of the current article illustrates how a high-low-high gradient in the general direction of HG suggested by Formisano et al. (2003) is also achievable in most variations of the common pattern if the gradients are positioned selectively into the frequency response pattern. Furthermore, as has been pointed out before (Langers and van Dijk, 2012), most of the studies preceding Formisano et al. (2003) showed a tendency for medial high frequency clusters to be situated just anterior to HG (in the first temporal sulcus) or posterior (in Heschl's sulcus) and low frequency areas to be on lateral crest of the HG. This is precisely what the pattern in Figure 3 predicts.

Concluding that there is a frequency response pattern that accommodates the previous functional data well, how are the tonotopic gradients positioned within this framework? And more importantly, how does the scheme of auditory fields that is well established in other primates fit into the human functional organization? While most human tonotopy studies emphasize the similarities of their gradient and field model to the non-human primate scheme, the range of different interpretations [with the extremes of Formisano et al. (2003) and Humphries et al. (2010)] suggest that a clear concept of this scheme, and how it can be applied to the human homolog, is lacking. Based on our experience in generating tonotopic maps in macaques in the context of the macro-anatomy of the individual animal, we will highlight a few often overlooked features of the non-human primate auditory cortex that help in understanding the relationship of functional and structural anatomy in humans and monkeys. These comparative insights highlight how surprisingly close the human tonotopy pattern is to that in non-human primates in most aspects, providing further justification for the attempt to find a similar field organization framework in humans that has proven so useful in other primates.

The first feature we look at is the direction of the tonotopic axis defining fields A1 and R in monkeys. In contrast to the most frequent interpretation in the human tonotopy studies this axis shows neither a simple posterior-anterior direction nor are the two respective gradients strictly collinear. As outlined above, non-human primates demonstrate gradients that progress from medial high frequency maxima to a more laterally located low-frequency maximum leading to a kink in the tonotopic axes. As we can see in Figure 3B, human and non-human primates share this feature and reveal a very similar frequency response pattern. The second aspect we want to highlight is the relationship of macro-anatomy and functional organization common to human and non-human primates. At the center of the human tonotopy debate is the orientation of the tonotopic gradients and the auditory core fields with respect to HG. A common understanding is that monkeys [in contrast to apes (Hackett et al., 2001)] do not feature a gyrus homologous to HG but rather a mostly flat superior temporal plane. This is a simplification at best in the case of the macaque. Even though this species does not feature proper gyrii, we consistently find a protuberance that separates the depth of the circular sulcus from the posterior planum. In addition to the anterior limitation of the circular sulcus this protuberance is in some cases additionally limited by a further groove (Intercalated sulcus) in the posterior planum forming a mini-gyrus (Annectant gyrus) which is located in the center of the auditory core (Jones et al., 1995) (Figure 3B). We postulate that the circular and intercalated sulci define a precursor to human HG. As discussed above this HG-like protuberance is a good predictor for the low frequency response maximum, a feature that it shares with the human HG (Figure 3B). Given these homologies in functional pattern and anatomical features it should be straightforward to identify the human homologs of the auditory core fields in the human superior temporal plane. The human homolog of A1 stretches from the posterior high frequency area in the vicinity of the medial HG in antero-lateral direction to the low frequency area on the crest of the HG (Figure 3). There it borders the human homolog of R which stretches from the crest into the depth of the first transverse sulcus in the same way the monkey R extends into the circular sulcus (the FTS is essentially an extension of the human circular sulcus). In conclusion, we find that the HG is a good predictor of the approximate location of the human core area but, in contrast to the classical *and* the perpendicular configuration, the core's orientation is in a rather oblique relationship to this prominent anatomical feature. Furthermore, the human scheme matches the non-human primate scheme in frequency response pattern, global gradient orientation and even in the relative position of the macro-anatomical features. Thus, we call this alternative model the “oblique configuration.” While this proposed model, similar to the perpendicular configuration, suggests that the human core area extends beyond the banks of the HG, this is limited to relatively small areas postero-medial and antero-lateral to the HG. Thus, the resulting pattern is very close to the results of the earlier cytoarchitectonic studies (Brodmann, 1909; von Economo and Koskinas, 1925; von Economo and Horn, 1930) and, given the individual variability in humans, it is only subtly different from and not necessarily irreconcilable with a core area confined to the middle portion of HG as suggested by other cytoarchitecture studies (Beck, 1928; Galaburda and Sanides, 1980; Rivier and Clarke, 1997; Morosan et al., 2001); see also (Figure 2).

Once the location of the auditory core fields is defined within the human tonotopic pattern, it is also straightforward to predict the human homologs of the belt fields by using the tonotopic reversals analogous to those that define the non-human primate scheme (Figure 3B, right). Furthermore, a number of the human tonotopy studies reported some evidence for additional low frequency reversals anterior and posterior of the high frequency areas of the common tonotopy pattern. The core-like field RT, caudal belt areas CM, and CL can be estimated with this additional information (Figure 3B). Taken together, an entire scheme of auditory functional fields in the superior temporal plane is derived that, as has been outlined above, shows a remarkable similarity to the situation in non-human primates in functional (frequency response) and anatomical terms. This similarity in the orientation of auditory fields and gradient axes across primates seems to us biologically more plausible than human core areas that deviate considerably in orientation from monkey homolog as suggested by the classical configuration and to some degree by the perpendicular configuration. Given that the perisilvian areas and superior temporal sulcus seem to share many response features across primates, a reorientation of auditory core and belt in respect to its immediate cortical environment would lead to a discontinuity at its borders that would be difficult to explain in evolutionary terms.

## Outstanding issues

The evidence above based on tonotopy suggests a clear organization of human core areas within the auditory cortex. There is an immediate need to reconcile models in which core extends beyond HG and cytoarchitectonics or staining studies in which the core is largely confined to HG. This effort is hampered by intersubject variability and the absence of studies of tonotopy and anatomy in the same subjects. Also potential alternative approaches to functionally delineate core-belt borders have not yet been calibrated to the anatomical definitions of core areas because the traditional staining techniques can only be applied to post-mortem brains. New methods to allow “*in vivo* cytoarchitechtonics” in human and non-human primates have the potential to achieve this. For example, (quantitative) T1 mapping (Bock et al., 2011; Glasser and Van Essen, 2011) seems particularly promising to us because it allows the definition of more heavily myelinated core cortex, a feature that was first mapped in the anatomical studies of Flechsig (1920).

Further work is required to better establish the organization of areas beyond the core, possibly by applying novel stimuli that drive belt areas better than the narrowband stimuli used for tonotopic mapping. In general, remaining differences in frequency response pattern and gradient locations across the cortex might be addressed by agreeing on standard practices for data co-registration (including flat-mapping) and gradient quantification.

The goal of this work is a robust scheme for the definition of functional areas in humans that might in future properly justify the application of primate nomenclature to human studies and allow the development of better-defined primate models for human auditory cognition. Here we suggested a unified primate model of core and belt fields which provides testable hypotheses for future functional and anatomical comparative studies in primates.

### Conflict of interest statement

The authors declare that the research was conducted in the absence of any commercial or financial relationships that could be construed as a potential conflict of interest.
